# Growth of Quasi-Two-Dimensional CrTe Nanoflakes and CrTe/Transition Metal Dichalcogenide Heterostructures

**DOI:** 10.3390/nano14100868

**Published:** 2024-05-16

**Authors:** Dawei Cheng, Jiayi Liu, Bin Wei

**Affiliations:** School of Materials, Sun Yat-sen University, Shenzhen 518107, China; chengdw5@mail2.sysu.edu.cn (D.C.); liujy396@mail2.sysu.edu.cn (J.L.)

**Keywords:** CVD, quasi-two-dimensional CrTe, WSe_2_, MoS_2_, heterostructures

## Abstract

Two-dimensional (2D) van der Waals layered materials have been explored in depth. They can be vertically stacked into a 2D heterostructure and represent a fundamental way to explore new physical properties and fabricate high-performance nanodevices. However, the controllable and scaled growth of non-layered quasi-2D materials and their heterostructures is still a great challenge. Here, we report a selective two-step growth method for high-quality single crystalline CrTe/WSe_2_ and CrTe/MoS_2_ heterostructures by adopting a universal CVD strategy with the assistance of molten salt and mass control. Quasi-2D metallic CrTe was grown on pre-deposited 2D transition metal dichalcogenides (TMDC) under relatively low temperatures. A 2D CrTe/TMDC heterostructure was established to explore the interface’s structure using scanning transmission electron microscopy (STEM), and also demonstrate ferromagnetism in a metal–semiconductor CrTe/TMDC heterostructure.

## 1. Introduction

The discovery and development of novel two-dimensional materials and their heterostructures are critical to next-generation nanodevices in the post-Moore’s law era, with materials exhibiting unique physical properties such as magnetism [1,2,3], ferroelectricity [4,5,6], or high carrier mobility [7,8,9], which can serve as the foundation for multifunctional devices such as photoelectronic and fast transistors, which are of particular interest [10,11,12,13]. Additionally, the integration of various types of two-dimensional materials has become an effective way to engineer interfaces or electronic structures. New functionality can arise from a well-designed interface between two dissimilar materials, creating novel phenomena such as quantum wells [14] or superlattices [15] and opening up new research fields.

As a quasi-2D material possessing intrinsic ferromagnetism, CrTe emerges as an excellent candidate for next-generation spintronics and electronics and has been widely researched due to its low-dimensional magnetism [16,17,18,19,20,21]. However, fully exploiting the potential of its applications requires heterostructure coupling to improve its properties and performance. For example, the Curie temperature of bulk CGT (61K) is increased considerately when in the form of a Bi_2_Te_3_/Cr_2_Ge_2_Te_6_ (CGT) heterostructure [22]; the interfacial atom-reconstruction-induced Moiré super crystal is found in a Cr_5_Te_8_/WSe_2_ heterostructure [23]; the coexistence of ferroelectricity and ferromagnetism was found in an atomically thin 2D Cr_2_S_3_/WS_2_ vertical heterostructure [24]; and a performance-enhanced photodetector was fabricated based on a MnTe/WS_2_ heterostructure [25]. These novel and fascinating properties induced by heterostructures, along with their enhancement of device performance, broaden the application range of two-dimensional materials and inspire researchers to design and integrate various functional modules from different perspectives to make the best of individual materials. While CrTe and its corresponding heterostructure systems possess rich physical properties, the Cr_x_Te_y_ family includes numerous phase structures with different chemical stoichiometries [17,19,26,27,28,29]. Additionally, unlike graphene, the conventional mechanical exfoliation method is ineffective for preparing CrTe [30,31,32,33] due to its quasi-2D non-layered structural characteristics, and CrTe products synthesized by chemical methods frequently suffer from impurities. Hence, the development of an efficient and facile method for preparing quasi-2D heterostructures is crucial for advancing the application of functional 2D devices.

As a mature industrial method, chemical vapor deposition (CVD) provides a general methodology for growing non-layered materials and their heterostructures [34]. So far, there have been several precedent-setting examples of quasi-2D nonlayered material synthesis, such as CrSe [35], Fe_7_Se_8_ [36], MnSe [37], and Cr_2_S_3_ [38]. Based on these materials, heterojunctions can be constructed on transition metal dichalcogenides (TMDC) or other 2D material templates using a two-step CVD method. These fabricated heterojunctions, such as CrSe_2_/WSe_2_ and Cr_5_Te_8_/WSe_2_, exhibit fascinating phenomena, including valley polarization and proximity effects, due to interface coupling [23,39]. By controlling various parameters such as the temperature or the precursor mass ratio, the morphology and phase can be tuned, ultimately yielding high-purity and high-crystalline-quality products. According to these reported works [40,41,42,43], it is evident that the CVD method is efficient and capable of obtaining single crystal 2D or quasi-2D materials and their heterostructures.

In this work, we report a selective one-/two-step growth method for high-quality single crystal CrTe nanoflakes and their TMDC-coupled heterostructure by adopting a universal CVD strategy with the assistance of molten salt and mass control. The as-grown nanoflakes and heterostructures were characterized using various methods to identify the phase, structure, and elemental composition. Scanning transmission electron microscopy was used to investigate the interface of the heterostructure in depth and to reveal the interfacial structures of nonlayered and layered heterostructures at an atom-scale resolution. The properties of CrTe and its heterostructure were explored using magnetic and electrical measurements. Our work opens up a door for growing specific phases of quasi-2D materials and their epitaxial heterostructures, which facilitate the realization of a platform used to explore and regulate the effects in the system and promote their application opportunities in future multifunctional nanodevices.

## 2. Materials and Methods

### 2.1. Preparation of Single-Crystal CrTe Nanoflakes

**Figure 1:**
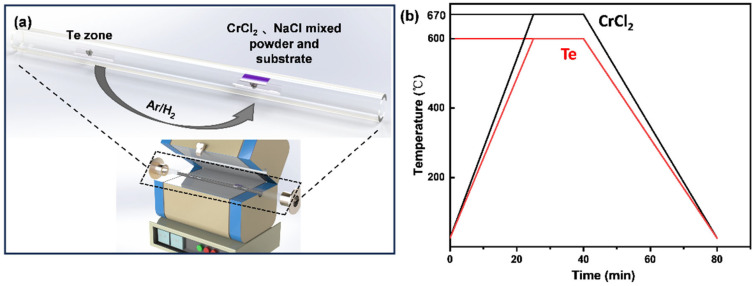
Schematic illustration of high-quality single-crystal CrTe nanoflakes growth. (**a**) Custom-made CVD furnace with a quartz tube inside and diagram of salt cosolvent reaction for growth of single-crystal CrTe; (**b**) schematic diagram of heating process for growth of CrTe nanoflakes.

Quasi-2D CrTe single-crystal nanosheets were synthesized on SiO_2_/Si substrate (300 nm thick SiO_2_) or mica at atmospheric pressure in a CVD system equipped with a 1-inch quartz tube, as illustrated in Figure 1a. Chromium dichloride (CrCl_2_, Amethyer, 97%) and tellurium powder (Te, Macklin, 99.9%) were used as the Cr source and Te source, respectively. Specifically, 20 mg of CrCl_2_ and a small amount of sodium chloride (NaCl, Macklin, 99%) powder were mixed in a quartz boat, which was placed in the second heating zone of a furnace, downstream of the tube in the CVD system. The SiO_2_/Si substrate (or mica) was placed facedown onto the CrCl_2_ precursor. A quartz boat, loaded with 10 mg Te powder, was placed upstream in the first heating zone. Quick actions must be taken to avoid precursor degradation during the experiment since CrCl_2_ is super hydrolyzed in the air. Before starting, the quartz was evacuated, and then Ar gas was introduced for 3 min to purge the tube. Finally, 150 sccm Ar/H_2_ (10%) was used as a carrier gas to facilitate the reaction system. As shown in Figure 1b, the CrCl_2_ zone was heated to 670 °C within a 30 min ramping time, and the Te powder was heated to 500 °C within the same time frame. The temperature in each heating zone was maintained for 10 min to synthesize the products. After the reaction was completely finished, the lid of the tube furnace was opened and the furnace was rapidly cooled to room temperature, in order to obtain CrTe nanoflakes on the SiO_2_/Si (mica) substrate.

### 2.2. Preparation of CrTe/MoS_2_ Heterostructure

**Figure 2:**
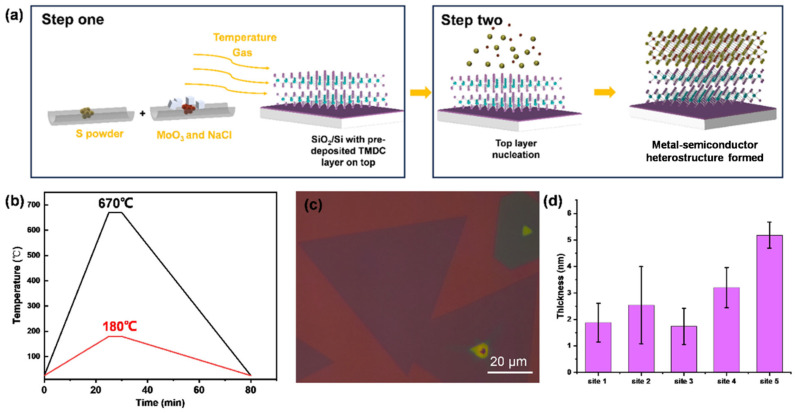
(**a**) The schematic diagram of reaction principle of two-step CVD method for preparation of CrTe-TMDC heterojunctions; (**b**) schematic diagram of heating process for growth of MoS_2_ nanoflakes; (**c**) optical image of prepared MoS_2_ template; (**d**) statistical result of thickness uniformity for MoS_2_ distributed on SiO_2_/Si substrate.

The CrTe-MoS_2_ heterostructure was fabricated using a two-step CVD method, as depicted in Figure 2. Ultrathin MoS_2_ flakes were pre-deposited using CVD on SiO_2_/Si substrate and then used as a template in the second CVD step. In the first step, 20 mg S powder and 10 mg MoO_3_, mixed with a small amount of NaCl powder, were loaded into the chemical boat and placed at upstream and downstream points, respectively. The temperature ramping process, as illustrated in Figure 2b, was undertaken; the MoO_3_ zone was heated to 670 °C within 25 min, while the S zone was heated to 180 °C, and both heating zones were kept at each temperature for 5 min. The SiO_2_/Si substrate was placed facedown onto the MoO_3_ precursor. To facilitate the reaction system, 150 sccm of Ar gas was used as a carrier gas. After the reaction was completely finished, the lid of the tube furnace was opened and the furnace was rapidly cooled to room temperature to obtain ultrathin MoS_2_ on the SiO_2_/Si substrate. In the second step, the method described in Section 2.1 for obtaining CrTe nanoflakes was repeated, with the exception that the clean substrate was replaced by a substrate with MoS_2_ pre-deposited on it. The relatively higher decomposition temperature of MoS_2_, compared to the synthesis temperature of CrTe, ensured the integrity of the whole heterostructure.

The CVD-grown MoS_2_ nanoflakes in the first step are displayed in Figure 2c; the large lateral size of these templates (up to ~100 μm without a dangling bond) allowed the precursor to migrate onto the surface and form good-quality vertical heterostructures. To assess the uniformity of the MoS2 nanoflakes on the substrate, we randomly selected five sites on the substrate and collected AFM thickness data from five nanoflakes at each site. A statistical plot is provided in Figure 2d to illustrate the thickness distribution. The result showed that the WSe_2_ on SiO_2_/Si exhibited relatively good homogeneity, with a thickness ranging from 2 to 5 nm.

### 2.3. Preparation of the CrTe-WSe_2_ Heterostructure

**Figure 3:**
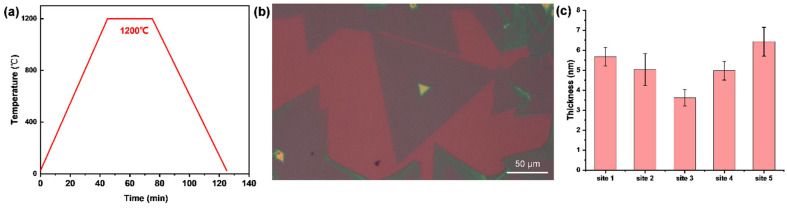
(**a**) Schematic diagram of heating process for the growth of WSe_2_ nanoflakes; (**b**) optical image of prepared WSe_2_ template; (**c**) statistical results of thickness uniformity for WSe_2_ distributed on SiO_2_/Si substrate.

The CrTe-WSe_2_ heterostructure was fabricated using a two-step CVD method. The WSe_2_ nanoflakes were grown on SiO_2_/Si using CVD in the first step; 1 g of WSe_2_ powder (Macklin, 99.9%) was loaded into the chemical boat at the center of the quartz tube, and two pieces of SiO_2_/Si substrate (300 nm SiO_2_) were placed, faceup, 15 cm away from the source. The WSe_2_ zone was heated to 1200 °C within a 45 min ramping time, and this temperature was maintained for 20 min for deposition (Figure 3a). To facilitate the reaction system, 200 sccm of Ar gas was used as a carrier gas. After deposition, the lid of the tube furnace was opened and the furnace was rapidly cooled to room temperature to obtain thin WSe_2_ flakes on the SiO_2_/Si substrate (Figure 3b). In the second step, the method described in Section 2.1 for obtaining CrTe nanoflakes was repeated, with the exception that the clean substrate was replaced by a substrate with WSe_2_ pre-deposited on it. The statistical results of WSe2 thickness, as shown in Figure 3c, indicated that the prepared WSe_2_ nanoflakes had a relatively uniform thickness distribution of 4–7 nanometers.

### 2.4. Characterization Methods

The HAADF-STEM images were obtained using an FEI Titan Themis equipped with an aberration corrector, whose resolution could reach the sub-Å level. The cross-sectional samples of CrTe/WSe_2_ and CrTe/MoS_2_ heterostructures were prepared using the focused ion beam (FIB) technique. The area of interest within the heterostructure was identified by SEM, and then a protective layer (Pt layer) was deposited onto the sample to protect the sample surface from the ion beam during the cross-sectioning process. A cross-sectional lamella was then cut using the ion beam, and the lamella was lifted out by a manipulator tip in order to attach everything to the TEM grid via Pt deposition. Finally, the cross-section underwent thinning and polishing for HAADF-STEM measurements.

An optical microscope (OM) and a scanning electron microscope (SEM, Hitachi, SU5000, 20 kV, working distance 13 mm, Hitachi High-Tech Corporation, Tokyo, Japan) were used to observe the morphology of the nanosheet samples by taking secondary electron images. The microscope was equipped with a related detector for energy dispersive spectroscopy (EDS, Bruker, XFlash6106, Billerica, MA, USA) and electron backscatter diffraction (EBSD, eFlash XS, Oxford Instruments plc, Abingdon, UK) for structural and elemental analysis. In addition to EDS, X-ray photoelectron spectroscopy (XPS, ESCALAB 250Xi, Thermo Fisher Scientific Inc., Waltham, Massachusetts, USA) was used complementarily to identify the constituent elements.

**Figure 4:**
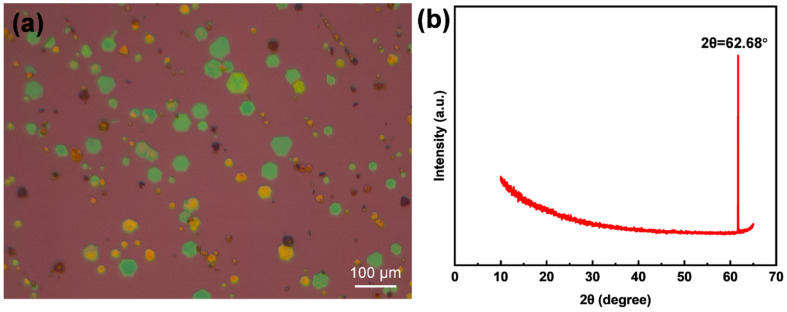
Local optical images of SiO_2_/Si substrate used for XRD measurement with CrTe nanoflakes randomly distributed (**a**) and XRD pattern of the bare SiO_2_/Si substrate (**b**).

X-ray diffraction (XRD) measurements were conducted using the BRUKER D8 ADVANCE (Bruker Corporation, Billerica, MA, USA), equipped with Cu Kα radiation. The flake samples directly grown on the substrate using CVD are shown in Figure 4a, covering a ~10% area of the substrate surface. The pattern of the bare SiO_2_/Si substrate, which shows a single peak at 62.68° in Figure 4b, was collected first to eliminate the interference from the substrate.

An atomic force microscope (AFM, HORIBA AIST Smart SPM, Kyoto, Japan) was used to measure the thickness and morphology of the grown nanoflakes. The samples measured were all grown on SiO_2_/Si or mica substrate.

Raman spectra were obtained using a Raman spectrometer, the Horiba XploRA PLUS Scientific (Kyoto, Japan). A green laser with a 532 nm wavelength was used as an excitation source. The Raman signal from SiO_2_/Si was not within the range of the measurement parameters.

For the electrical and magnetic properties, electrical measurements were performed at room temperature in a dark environment and characterized using a semiconductor parameter analyzer (Keithley 4200A, Cleveland, OH, USA), and the magnetization studies were carried out from 5 to 300 K using the VSM module of the Quantum Design Physical Property Measurement System (Quantum Design PPMS DynaCool, San Diego, CA, USA) equipped with a low-temperature platform.

### 2.5. Device Fabrication

The devices were fabricated by employing standard e-beam lithography and a metal deposition process. After the patterns were transferred onto substrates with photoresists, metal bilayers with 10 nm Ti and 60 nm Au were deposited by thermal evaporation and used as contact electrodes. Finally, the substrate with devices was immersed in acetone solution, and the excess metal and photoresist were removed using the lift-off process.

## 3. Results and Discussions

### 3.1. Characterization of CrTe Single Crystal

Analysis of the CVD-grown CrTe method: During the CVD process, the molten salt medium played an important role in forming the target CrTe product. Once the salt was introduced, it helped to lower the melting point of CrCl_2_ and was also involved in the reaction process as a cosolvent system, in order to lower the nucleation barrier energy of the 2D single crystals [35]. We first attempted, but failed, to obtain thin flakes without the assistance of salt; in this situation, CrTe tends to grow in a 3D island mode, i.e., only in a vertical direction, resulting in bulky particles or thicker films. 

**Figure 5:**
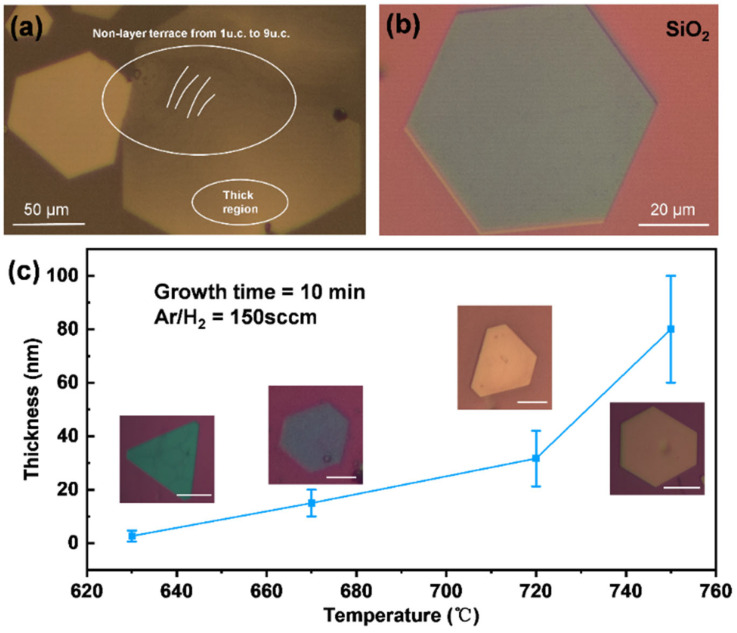
Optical images of large-sized CrTe single crystals on mica (**a**) and SiO_2_/Si (**b**) substrates and (**c**) evolution of flake thickness and shape at varying growth temperatures from 620 to 750 °C.

Some 2D nonlayered materials are suitable for growth on mica due to the suspended bonding-free surface; however, CrTe nanoflakes have great compatibility with several substrate options. Figure 5a,b show optical images of large-sized hexagonal-shaped CrTe nanoflakes on mica and SiO_2_/Si substrates, and both show smooth surface morphologies without the adherence of impurities or microscopic defects. It is interesting that a clear layered hierarchy, from 1 to 9 unit cells thick, is displayed on the mica substrate. Therefore, the proposed CVD method is highly suitable and efficient for the growth of CrTe single crystals.

To further optimize the growth parameters, systematic results were collected to reveal the effect of temperature on the thickness and shape evolution of CrTe nanoflakes (Figure 5c). With the other parameters, including growth time and gas flow rate, which remained unchanged, CrTe nanoflakes synthesized at various temperatures exhibit notable thickness and shape differences, as summarized in Figure 5c, where the increase in flake thickness can be observed from the change in flake color. Specifically, the average thickness of the flakes increases from 3 nm to 80 nm as the growth temperature increases from 620 to 750 °C, accompanied by the evolution of flake shape from truncated triangle to perfect hexagon. In fact, the growth time, mass ratio of precursors, and gas flow rate all have specific impacts on the growth of CrTe crystals; they can be tuned by adjusting the synergistic effect of thermodynamic and kinetic factors.

**Figure 6:**
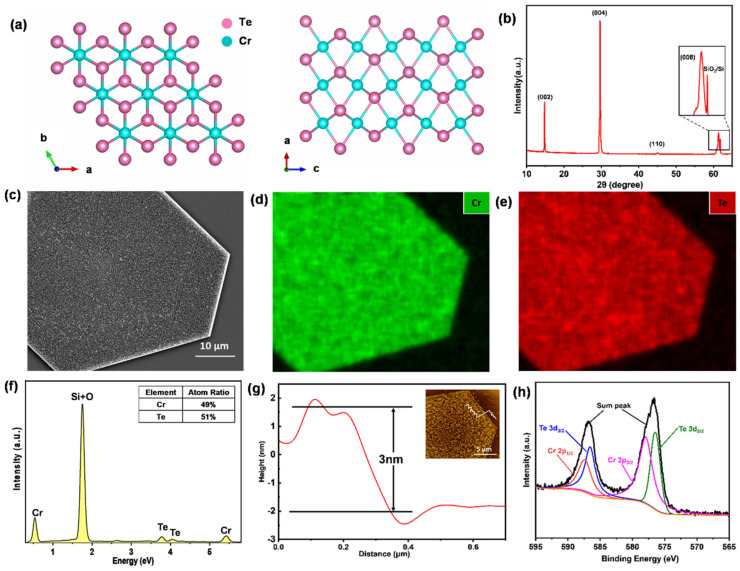
The characterizations of CrTe nanoflakes. (**a**) Top and side view of atomic structure model of CrTe; (**b**) XRD patterns of CrTe flakes peaking at four noticeable positions; (**c**–**e**) SEM images and corresponding EDS mappings of CrTe flakes; (**f**) energy dispersive spectrum and quantitative result of CrTe nanoflakes; (**g**) AFM image showing ultrathin CrTe flake with a thickness of about 3 nm (inset: the AFM morphology image of CrTe flakes); (**h**) high-resolution XPS scanning of Cr 2p and Te 3d orbit of CrTe single crystal.

Structural and compositional analysis: Figure 6a shows the top and side view of the atom structure configuration for the CrTe crystal, whose lattice adopts a NiAs hexagonal structure, which belongs to the P63/mmc space group with lattice parameters of a = b = 0.3978 nm and c = 0.6228 nm. Each Cr atom is surrounded by six Te atoms. Analogous to layered CrTe2, the non-layered Cr_x_Te_y_ is a derivative compound of the CrTe2 “backbone” structure, which is self-intercalated with a Cr atom layer in van der Waals spaces. The classifications of all types of Cr_x_Te_y_ were identified using the percentages and sites of occupied Cr atoms in the intercalated layer.

To study the atomic structure and crystalline qualities of an as-grown product, XRD patterns were collected to demonstrate the phase and crystallinity. The result (Figure 6b) shows an additional substrate peak and four main peaks of synthesized CrTe nanoflakes, with an average thickness of around 20 nm, on the SiO_2_/Si substrate, corresponding to the (002), (004), (110), and (008) lattice planes of the hexagonal crystal structure. The sharp peaks and narrow half-peak width indicate that the grain size is large and there is a high level of crystallization. In addition, the low-magnification SEM image (Figure 6c) and corresponding EDS mapping (Figure 6d,e) displayed clean and smooth surface conditions and homogeneous element distribution throughout the entire sample, and the quantitative result indicated that CrTe flakes possessed a stoichiometric ratio of 1:1 for Cr and Te elements (Figure 6f). XPS was further used to confirm the target’s element composition and chemical state (Figure 6h); the fitted result showed that the bonding energies of Te 3d_3/2_ and 3d_5/2_ are 586.58 eV and 576.38 eV, and that those of Cr 2p_1/2_ and Cr 2p_3/2_ peak at 587.48 eV and 577.98 eV, respectively. The bonding energies of electrons in the Te and Cr atoms are higher than in the usual chemical state for CrTe, which were reported to be 582.0 eV and 572.0 eV for the Te 3d_3/2_ and 3d_5/2_ orbit, and 586.0 and 576.0 eV for the Cr 2p_1/2_ and 2p_3/2_ orbit, suggesting that the surface chemical environment changes as tellurides are generally sensitive to air exposure [26,44,45], which causes the bonding energy to increase. The height of ultrathin nanoflakes was scanned using AFM, and it was found to be 3 nm thick after deducting the substrate signals, as shown in Figure 6g. Based on the analysis above, we have prepared a CrTe single crystal with high levels of crystallinity and surface quality using the molten salt-assisted CVD method.

### 3.2. Characterization of CrTe-WSe_2_ Heterostructure

**Figure 7:**
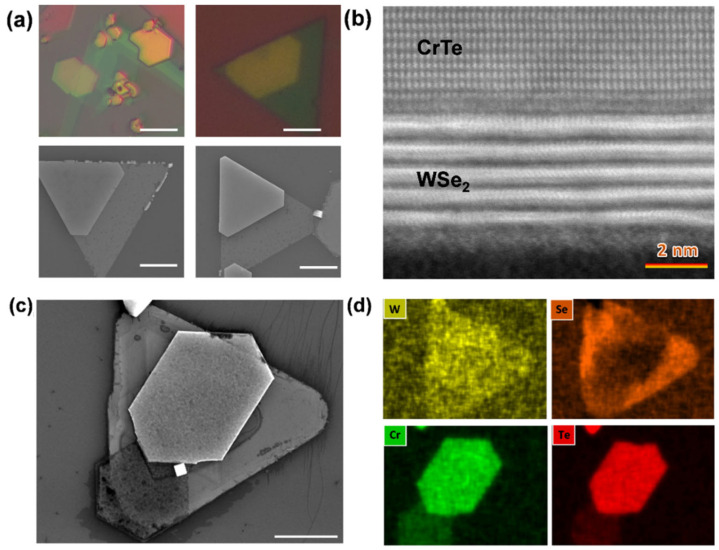
The microscopic characterizations of CrTe/WSe_2_ heterostructure. (**a**) Optical and SEM images of CrTe/WSe_2_ heterostructures; (**b**) cross-sectional HRSTEM image of CrTe/WSe_2_ heterostructure; (**c**,**d**) SEM images of CrTe/WSe_2_ heterostructures (**c**) and its corresponding EDS mapping (**d**). (Scale bar in (**a**,**c**): 5 μm).

To explore the principle of nonlayered material/layered TMDC-coupled heterostructure systems and the level of lattice mismatch from an atomic perspective, a CrTe-WSe_2_ heterostructure was chosen as the object of investigation. As shown in the OM and SEM images in Figure 7a,c, the CrTe crystal grows epitaxially on the WSe_2_ template and aligns with the edge of the WSe_2_ crystal, forming a hybrid heterostructure with layered and non-layered material. The EDS mapping of the CrTe-WSe_2_ heterostructure also shows a uniform elemental distribution throughout the entire sample (Figure 7d). The lattice mismatch between CrTe and WSe_2_ is calculated to be ~22%; however, the epitaxial growth of a good-quality vertical heterostructure is still present due to the weak van der Waals (vdW) interaction at the interface. To gain more detailed information, the HAADF-STEM image of the cross-sectional heterojunction is shown in Figure 7b, wherein the distinct phase differences can be observed from the atomic structure. The vdW structure of WSe_2_ can be seen through the bottom layer of the atomic arrangement, while the upper layer corresponds to the (120) lattice plane of single-crystal CrTe, and there is a sharp interface in between the two, without obvious defects compared to some other mechanical stacking heterojunctions.

**Figure 8:**
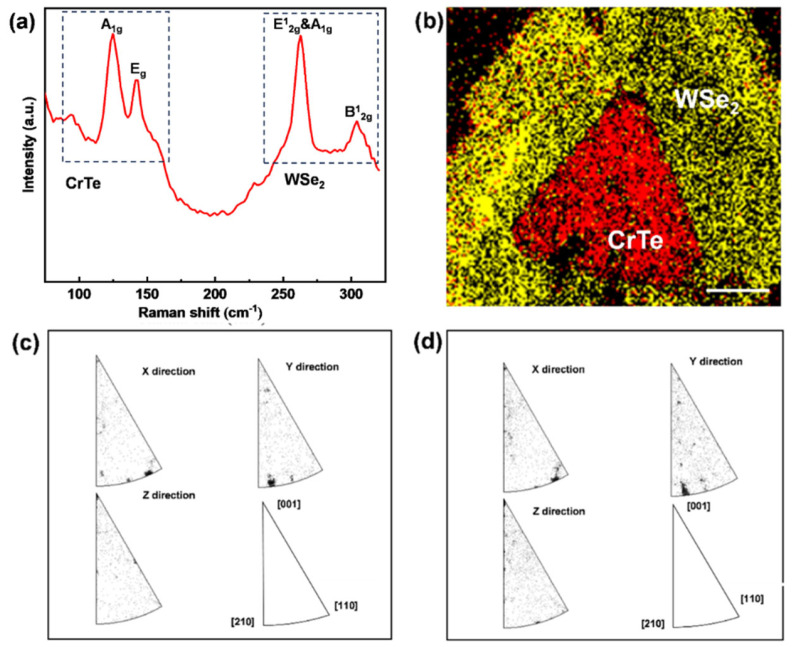
The Raman and EBSD characterizations of the CrTe/WSe_2_ heterostructure. (**a**) Raman spectra of CrTe and WSe_2_ vibration modes; (**b**) EBSD mapping of WSe_2_ and CrTe phases in heterostructure; (**c**,**d**) inverse pole figures for samples in the x-, y-, and z-axis directions of WSe_2_ (**c**) and CrTe (**d**), respectively. (Scale bar: 6 μm).

In addition to the microscopic characterizations, Raman spectra and EBSD are used to reveal the lattice vibrations and spatial orientations of both materials in the heterostructure. The Raman spectra of CrTe-WSe_2_, as shown in Figure 8a, indicate the coexistence of chemical vibrations in both the CrTe and WSe_2_ crystals. The spectra of CrTe nanoflakes peak at two main characteristic positions, 117 cm^−1^ and 137 cm^−1^, corresponding to the out-of-plane A_1g_ and in-plane E_g_ vibration modes, respectively. The peaks at 263 cm^−1^ and 304 cm^−1^ correspond to the E12g, A_1g_, and E^1^_2g_ vibration modes of few-layer WSe_2_. Figure 8b shows the EBSD phase mapping of CrTe-WSe_2_, with a uniform phase structure. Figure 8c,d show the inverse pole figures of one sample of the CrTe-WSe_2_ heterostructure, in the x-, y-, and z-axis directions, extracted from the EBSD results. The pole points appear in almost the same directions in two inverse pole figures, which indicates that the WSe_2_ and CrTe layers possess the same spatial orientation. Additionally, both WSe_2_ and CrTe belong to the same P63/mmc space group. This special alignment mitigates the impact of crystal mismatch and raises the possibility of the formation of such hybrid heterostructures.

### 3.3. Characterization of the CrTe-MoS_2_ Heterostructure

In the CrTe/TMDC heterostructure system, the bottom van der Waals layers serve as a template with atomically smooth surfaces and without dangling bonds, which is favorable for the subsequent CVD epitaxy of non-layered CrTe flakes. The typical TMDC material, MoS_2_, and WSe_2_ are similar with regard to their atom configuration; therefore, a high-quality heterostructure can be achieved, based on both WSe_2_ and MoS_2_ templates.

**Figure 9:**
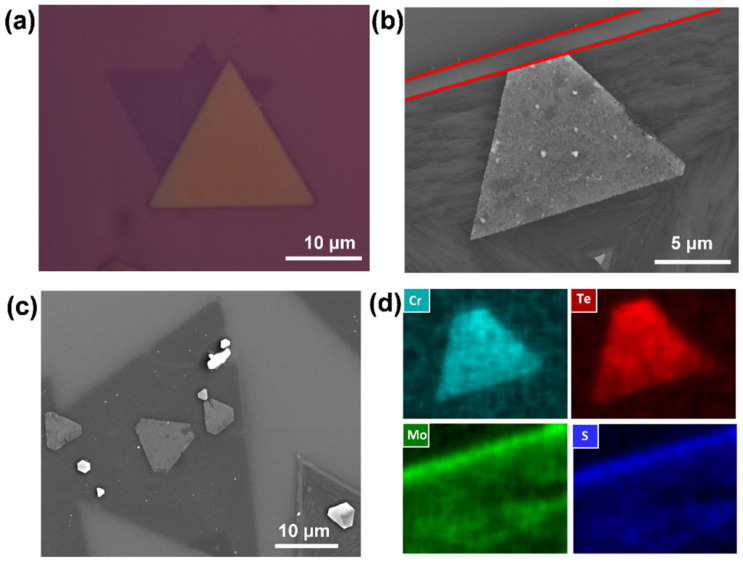
The characterizations of the CrTe/MoS_2_ heterostructure. (**a**–**c**) Optical and SEM images of CrTe/MoS_2_ heterostructure; (**d**) EDS elements mapping, corresponding to (**b**). (The red lines: the frame edge of a core-shell type MoS_2_ nanoflakes).

The experimental results are consistent with our expectations. Similar morphologies are shown in the optical and SEM images in Figure 9a–c, demonstrating an obvious bilayer heterostructure with different shapes. We noticed that, when MoS_2_ forms a triangle frame at the edge which is slightly higher than the middle section of the MoS_2_ layer (the core–shell structure appears during the synthesis of the 2D TMDC material [46]), as marked by red lines in Figure 9b, the CrTe tends to be not only parallel to the bottom layer but oriented along the frame, forming a heterostructure in both the lateral and vertical directions. Again, the EDS mapping (Figure 9d) proves that the two-step CVD method is effective in synthesizing a CrTe crystal and its heterostructures.

**Figure 10:**
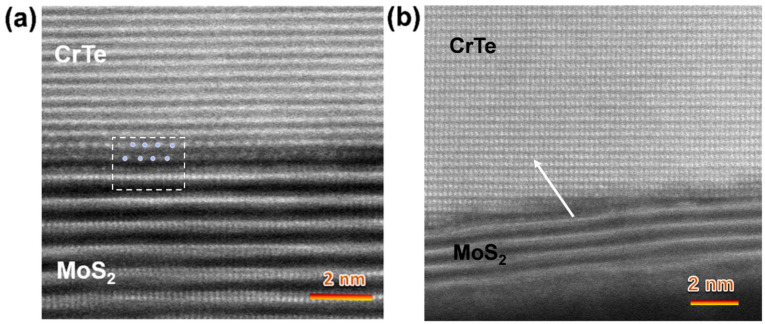
Cross-sectional high-resolution STEM image of CrTe/MoS_2_ with parallel orientation (**a**) and misaligned orientation (**b**). (The grey dot in a denotes Te atoms; dashed square: the interface region of CrTe/MoS_2_ heterostructure).

The cross-sectional HRSTEM images were also used to investigate the atomic arrangement at the interface. In Figure 10a, the lattice plane of the CrTe crystal presented in [110] and the atom denoted by grey dots with a brighter contrast are Te atoms; this indicates that the interface of CrTe and MoS_2_ consists of Te-S atom layers. However, it has been reported previously that the interface of the Cr5Te8/WSe2 heterostructure is reconstructed by a Cr atom layer [23]. Although these two heterojunctions have similarities in structure, they may take different routes to grow.

Interestingly, despite the parallel growth mode of the heterostructure, we also found that these two materials can be coupled together by forming a terraced interface structure (Figure 8b). In this situation, the CrTe capping layer stacks while rotating to an angle according to the MoS_2_ layers; the interface structure forms atomic-scale steps and severe crystalline lattice distortion may happen in this area. The CrTe layer lies on the MoS_2_ “slope” resulting from the misaligned orientation between the two individual heterolayers, and the structure of the crystal boundary alone (see arrow) evolves from a defective phase, with a lattice distortion region, to the normal CrTe crystal lattice.

### 3.4. Property Measurements

**Figure 11:**
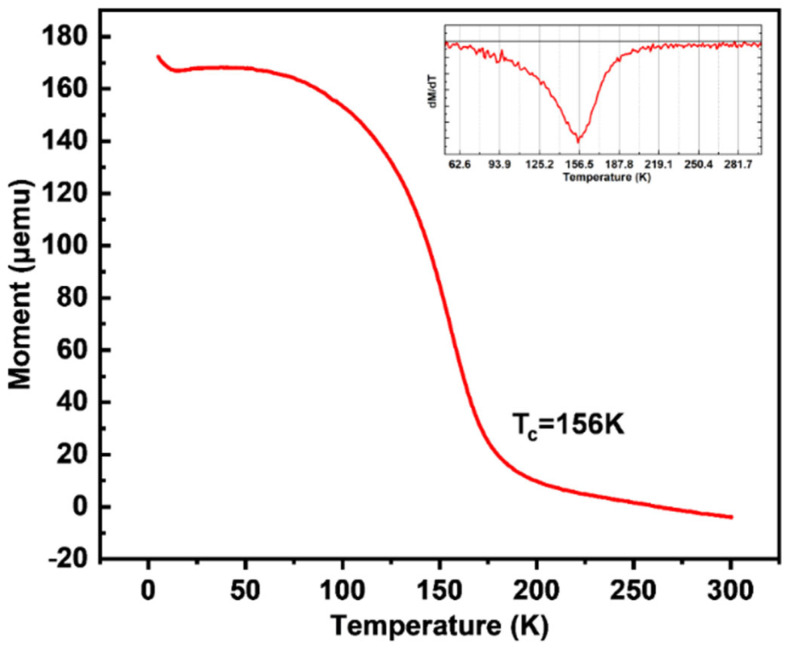
The temperature-dependent ZFC magnetic susceptibility of CrTe/MoS_2_ samples with an external magnetic field applied perpendicular to the c axis. (Inset: differential curve of thermos–moment curve).

The measurement of the magnetic properties of CrTe/MoS_2_ samples and how they change with temperature was performed by employing the VSM module in PPMS. The temperature-dependent zero-field-cooled (ZFC) magnetic susceptibility of CrTe-MoS_2_ samples with an external magnetic field perpendicular to the c axis was investigated. As shown in Figure 11, the sample exhibits typical ferromagnetic characteristics with a Curie temperature, T_c_, of ~156 K, wherein the differential curve of the thermos–moment curve reaches the extremum and the phase transition from ferromagnetism to paramagnetism is the most intense. Beyond 156 K, the thermal fluctuations disrupt the long-term magnetic order. In fact, the magnetism of CrTe/MoS_2_ originates from the magnetic CrTe layer.

Due to the randomness in the nucleation site of CrTe, we can find a CrTe-WSe2-CrTe three-layer structure on the substrates. Such a naturally grown heterostructure, with fewer defects at the interface, is a perfect physical model of a magnetic tunnel junction device, with CrTe as a magnetic electrode and WSe_2_ as a space layer. When the magnetization states of both electrode materials are parallel, a large tunnel current is generated. The current is small when both electrode materials are in an antiparallel state [47,48,49]. The ferromagnetism in this heterojunction implies significant potential for spintronics applications, such as spin valves and giant magnetic resistance devices [50,51].

**Figure 12:**
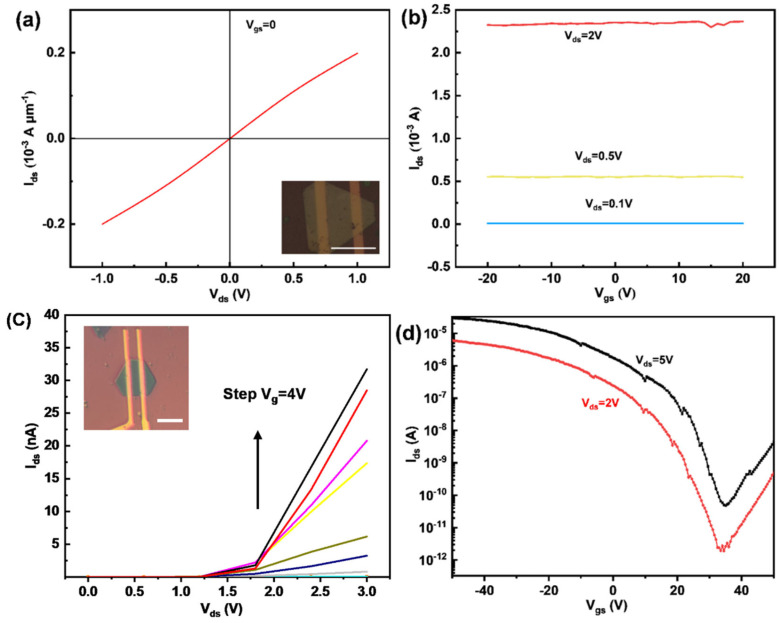
The electrical measurements of CrTe and WSe_2_. (**a**,**b**) The output and transfer characteristic curves of CrTe device, indicative of its metallic nature (inset: OM image of CrTe device, scale bar: 8 μm); (**c**,**d**) the output and transfer characteristic curves of WSe_2_ device, indicative of its semiconductor nature, the step size of V_gs_ for each curve is 4 V. (inset: OM image of WSe_2_ device, scale bar: 5 μm).

To verify the metal–semiconductor contact of CrTe-WSe2, further electrical measurements of CrTe and WSe_2_ were conducted. A field-effect transistor (FET) device was fabricated using a 10 nm thick CrTe nanosheet as a channel material, as shown in Figure 12. Figure 12a,b demonstrate the output and transfer characteristic curves of the CrTe device. A linear I_ds_-V_ds_ curve at room temperature indicates ohmic contact between CrTe and the metal electrodes. The results reveal that the conductance of CrTe is higher than that of the common 2D semiconductor material, including MoS_2_ and WSe_2_. [10,52], which manifests the metal conductive nature of the CrTe crystal and is also evidenced by the non-field-effect modulation in CrTe-based devices, as shown in Figure 12b. WSe_2_ is one of the most frequently used 2D semiconductor materials in nanodevices and shows promising potential for applications in electronics. Depending on the number of layers, WSe_2_ shows an indirect bandgap in its bulk state and a direct bandgap in its monolayer state. The bandgap of WSe_2_ is also thickness-related [53]. Figure 12c,d demonstrate the transfer and output characteristic curves of the WSe_2_ device. Based on the transfer curves in the graph and due to the relatively large work function of Ti metal, the WSe_2_ device tends to exhibit bipolar conductivity, with a high on/off ratio of approximately 10^6^ in the p-region and a minimum dark current reaching a magnitude of 10^−11^ A. The evident non-linear behavior of the output characteristics, resulting from the WSe_2_-Ti contact, indicates the semiconductor nature of WSe_2_.

Based on the analysis, the constructed CrTe-WSe_2_ is a 2D-material-based metal–semiconductor heterostructure. From this perspective, such a heterojunction could have the potential to be used in electronic applications as a sharp and naturally formed heterojunction interface [54], in order to reduce resistance caused by the Schottky barrier, which may be attributed to metal-induced gap states [55] and lithography [56] or metal deposition processes [57].

## 4. Conclusions

Ultrathin quasi-2D CrTe nanoflakes and CrTe/TMDC heterostructures were synthesized via a one-step/two-step molten salt-assisted CVD method. Various characterization methods, including microscopic and spectroscopic means, were used to confirm the structure, elemental composition, and crystallinity of as-grown nanoflakes and heterostructures. The interfacial structure and evolution of CrTe-TMDC heterostructures were explored using HRSTEM. The atomic arrangements reveal that the sharp interface is of high quality and that the Te-S chalcogenide atom layers make contact at the interface of CrTe/MoS_2_. The thermo-moment M-T curve indicates that the ferromagnetism in CrTe-MoS_2_ can persist up to ~156 K, showing its potential for spintronics applications. Moreover, the electrical properties of CrTe and WSe_2_ were investigated by fabricating devices to confirm the metal–semiconductor contact of CrTe-TMDC heterostructures. Our work provides an ideal platform for research on low-dimension heterostructures, their physical properties, and their possible applications in future spintronic and electronic devices.

## Data Availability

The data presented in this study are available upon request from the corresponding author.
